# Extensive herpes zoster involvement following mycophenolate mofetil therapy for sarcoidosis

**DOI:** 10.1007/s12348-011-0041-y

**Published:** 2011-09-25

**Authors:** Sandhya Hegde, Radha Annamalai, Jyotirmay Biswas

**Affiliations:** Medical Research Foundation, Sankara Nethralaya, Chennai, India

## Introduction

Sarcoid uveitis is usually a presumptive diagnosis based on the simultaneous presence of uveitis and clinicoradiographic or histological findings of sarcoidosis. Mycophenolate mofetil (MMF) is an anti-metabolite, selectively aimed at affecting lymphocytic action [1, 2]. It has been proven to be safe and effective in post organ transplant [3] and seems to have similar efficacy in non-infectious uveitis [4]. In patients who are corticosteroid resistant or require an unacceptable dose of corticosteroids to maintain remission, additional immunosuppression is used, including methotrexate, azathioprine, and MMF. We report an uncommon case of a 34-year-old male of sarcoid uveitis who developed extensive herpes zoster while on treatment with MMF.

## Case report

**Fig. 1:**
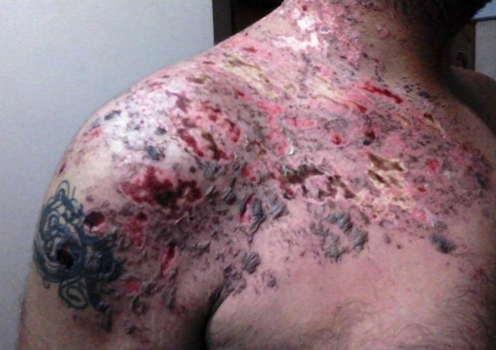
Clinical photograph showing extensive herpes zoster involvement in a 35-year-old patient

A 34-year-old male was referred to our hospital with a history of decreased vision as a result of recurrent bilateral posterior uveitis. He had repeated recurrences in spite of systemic corticosteroid therapy. At presentation to us, his best corrected visual acuity (BCVA) was 6/36, N36 in the right eye and 6/9, N6 in the left eye. Slit lamp examination revealed a normal anterior chamber and complicated cataract in the right eye. On fundus evaluation, both eyes revealed bilateral disc edema and vitritis and exudates in the macula in the right eye. He was not a known diabetic. Laboratory investigations for tuberculosis, collagen vascular diseases, and other infectious etiologies including human immunodeficiency virus were within normal limits except serum angiotension converting enzyme values which were borderline high. Treatment was initiated with MMF (2 g/day) [5] along with tapering doses of oral prednisolone (1 mg/kg/day) in view of the recurrent nature of the uveitis. The lesions resolved completely with treatment. He underwent cataract surgery in both eyes 6 months later under cover of MMF (1 g/day) and systemic corticosteroids. Postoperative period was uneventful. Four months later, he had a recurrence of fundus lesions and was started on oral prednisolone (60 mg/day) and his MMF dose was doubled to 2 g/day by his local ophthalmologist. He came to us within a month with extensive skin lesions over his right neck and upper thoracic region which was clinically diagnosed as herpes zoster (Fig. 1). MMF was stopped and oral acyclovir (400 mg five times per day) was started. With treatment, the herpetic lesions resolved and his ocular status was stable with BCVA 6/12, N12 in the right eye and 6/6, N6 in the left eye.

## Discussion

MMF is useful in various ocular inflammatory conditions. Its advantage is its potent corticosteroid sparing effect and relatively benign side effects such as gastrointestinal disturbances, alopecia, and transient leucopenia [6, 7].Our patient showed a good response to combined treatment with MMF (2 g/day) and prednisolone (1 g/day). But he developed extensive herpes zoster after 10 months of treatment requiring discontinuation of the drug. Opportunistic infections have been rarely reported in sarcoid uveitis in spite of the immune dysregulation noted in these patients [8]. Herpes zoster is known to occur in post organ transplant cases who are on multiple immunosuppressive drugs usually around the 9th to the 12th month as was seen in our patient [9]. But a multicentre randomized controlled trial of MMF in doses of 2–3 g/day for renal transplant patients did not report even a single case of herpes zoster [10]. Higher doses of MMF can have an atypical and disseminated varicella infection [11]. But herpes zoster as an opportunistic infection with oral steroids is rare and generally mild and self-limiting [12]. There are no reports of such extensive herpes lesions in otherwise immunocompetent individuals. Thus, in our patient, a cumulative immunosuppressive effect may have been the reason for such an extensive involvement with herpes zoster. A serology done prior to treatment is of no help as patients with varicella antibodies have also been found to develop full-borne varicella infection [13]. With discontinuation of MMF and appropriate anti-viral therapy, the lesions resolved completely. Studies like SITE study [14] have shown that the overall risk of opportunistic infections with the use of long-term immunosuppressives is very low. Our case highlights the fact that reactivation of herpes zoster can occur with therapeutic doses of MMF for uveitis in otherwise healthy immunocompetent adults. It does not necessarily prove that other such opportunistic infections may be a possible side effect with MMF treatment. It would be wise to enlighten the patients regarding this possibility and allow for early recognition and treatment.

## References

[1] Lipsky JJ (1996). Mycophenolate mofetyl. Lancet.

[2] Siepmann K, Huber M, Stubiger N, Deuter C, Zierhut M (2006). Mycophenolate mofetil is a highly effective and safe immunosuppressive agent for the treatment of uveitis: a retrospective analysis of 106 patients. Graefes Arch Clin Exp Ophthalmol.

[3] Sollinger HW (1995). Mycophenolate mofetil for the prevention of acute rejection in primary cadaveric renal allograft recipients. Transplantation.

[4] Thorne JE, Jabs DA, Qazi FA, Nguyen QD, Kempen JH, Dunn JP (2005). Mycophenolate mofetil therapy for inflammatory eye disease. Ophthalmology.

[5] Bhat P, Cervantes-Castañeda RA, Doctor PP, Anzaar F, Foster CS (2009). Mycophenolate mofetil therapy for sarcoidosis-associated uveitis ocular. Ocul Immunol and Inflamm.

[6] Ferreyra HA, Jayasundera T, Khan NW, He S, Lu Y, Heckenlively JR (2009). Management of autoimmune retinopathies with immunosuppression. Arch Ophthalmol.

[7] Zierhut M, Huber M, Stuebiger N, Deuter C, Siepmann K (2004) Mycophenolate mofetil (MMF) is a highly effective and safe immunosuppressive agent for the treatment of uveitis. Invest Ophthalmol Vis Sci 45:Abstract 3371. 2004 ARVO 10.1007/s00417-005-0066-8 16163494

[8] Girard N, Cottin V, Hot A, Etienne-Mastroianni B, Chidiac C, Cordier JF (2004). Opportunistic infections and sarcoidosis. Rev Mal Respir.

[9] Fuks L, Shitrit D, Fox BD, Amital A, Raviv Y, Bakal I, Kramer MR (2009). Herpes zoster after lung transplantation: incidence, timing, and outcome. Ann Thorac Surg.

[10] Anonymous (1996) A blinded, randomized clinical trial of mycophenolate mofetil for the prevention of acute rejection in cadaveric renal transplantation. The Tricontinental Mycophenolate Mofetil Renal Transplantation Study Group. Transplantation 61(7):1029–1037 8623181

[11] De D, Dogra S, Sharma A, Minz M, Handa S, Dutta A (2008). Persistent atypical varicella in two renal transplant patients and its relation to mycophenolic acid. Indian Journal of Dermatology,Venerology and Leprology.

[12] Wilcox CM, Schwartz DA (1992). A pilot study of oral corticosteroid therapy for idiopathic esophageal ulcerations associated with human immunodeficiency virus infection. Am J Med.

[13] Rothwell WS, Gloor JM, Morgenstern BZ, Milliner DS (1999). Disseminated varicella infection in pediatric renal transplant recipients treated with mycophenolate mofetil. Transplantation.

[14] www.med.upenn.edu/cpob/studies/studies_site.shtml

